# Blood pressure variability: a review

**DOI:** 10.1097/HJH.0000000000003994

**Published:** 2025-03-10

**Authors:** Spoorthy Kulkarni, Gianfranco Parati, Sripal Bangalore, Grzegorz Bilo, Bum Joon Kim, Kazuomi Kario, Franz Messerli, George Stergiou, Jiguang Wang, William Whiteley, Ian Wilkinson, Peter S. Sever

**Affiliations:** a Clinical Pharmacology Unit, Cambridge University Hospitals NHS Foundation Trust; b Department of Experimental Medicine and Immunotherapeutics, University of Cambridge, Cambridge, UK; c Department of Cardiology, IRCCS San Luca Hospiatal, Istituto Auxologico Italiano; d Department of Medicine and Surgery, University of Milano-Bicocca, Milan, Italy; e Bellevue Hospital Center and NYU School of Medicine, New York, New York, USA; f Department of Neurology, Asan Medical Centre, University of Ulsan College of Medicine, Seoul, Korea; g Division of Cardiovascular Medicine, Jichi Medical University School of Medicine, Tochigi, Japan; h University of Bern, Switzerland and Jagiellonian University, Krakow, Poland; i Hypertension Center STRIDE-7, University of Athens, Greece; j The Shanghai Institute of Hypertension, Shanghai, China; k Centre for Clinical Brain Sciences, University of Edinburgh, Edinburgh, UK; l National Heart & Lung Institute, Imperial College London, London, UK

**Keywords:** ASCOT study, blood pressure variability, coronary events, stroke

## Abstract

Blood pressure variability (BPV) predicts cardiovascular events independent of mean blood pressure. BPV is defined as short-term (24-h), medium or long- term (weeks, months or years). Standard deviation, coefficient of variation and variation independent of the mean have been used to quantify BPV.

High BPV is associated with increasing age, diabetes, smoking and vascular disease and is a consequence of premature ageing of the vasculature.

Long-term BPV has been incorporated into cardiovascular risk models (QRISK) and elevated BPV confers an increased risk of cardiovascular outcomes even in subjects with controlled blood pressure.

Long-acting dihydropyridine calcium channel blockers and thiazide diuretics are the only drugs that reduce BPV and for the former explains their beneficial effects on cardiovascular outcomes.

We believe that BPV should be incorporated into blood pressure management guidelines and based on current evidence, long-acting dihydropyridines should be preferred drugs in subjects with elevated BPV.

## BACKGROUND

Hypertension is the leading preventable cardiovascular (CV) risk factor. A systolic blood pressure (SBP) of ≥140 mmHg is associated with 14% of all-cause deaths [1]. In hypertensive patients, decisions on thresholds for treatment and on target blood pressure (BP) have traditionally been derived from observational studies and randomized controlled trials (RCTs) of drug treatment. There is overwhelming evidence the lower the achieved BP, the better the CV outcome. However, at most levels of BP, age is a more important determinant of risk. The relationship between BP and CV disease is also strongly influenced by the presence of comorbidities such as type 2 diabetes mellitus (T2DM), smoking and dyslipidaemia. However, residual risk remains even after the control of these risk factors raising the possibility that there are other important determinants of CV outcomes. Physiologically, BP is a continuous variable and is characterized by beat-to-beat variation as part of haemodynamic homeostasis. Variation in BP measured over 24 h results from complex interactions between the autonomic nervous system (ANS) and environmental stimulation including posture, exercise, emotion, circadian rhythms, and genetic factors unique to individuals. This is referred to as short-term BP variability (BPV). Longer-term BPV measured over days, weeks or months has emerged as a determinant of CV risk and is influenced by additional factors including vascular compliance and structure, use of antihypertensive drugs and their titration and adherence to treatment. For >20 years, it has been noted that BPV has been associated with the severity of end organ damage (EOD) including stroke, heart failure (HF), ischaemic heart disease (IHD), chronic kidney disease (CKD) and visual loss [2], with the longitudinal demonstration that increased 24 h BPV produced cardiac damage over a 7-year follow-up in treated hypertensives [3]. Despite mounting evidence, until recently, intra-individual visit-to-visit variability (VVV) of BP has been dismissed as random fluctuation around a patient's true underlying BP level. Interestingly, variability in other CV risk factors including fluctuation in weight, lipids [4] and blood sugar [5,6] also seem to have an important effect on CV outcomes. Variability in LDL-cholesterol and BPV may be synergistic and high variability in both measures is associated with a substantial high risk of CV events [7]. Others have shown similar synergistic relationships of glycemic, LDL-C, BPV and body weight variability on CV events [8]. Whether certain medications can reduce synergistic variability in more than one risk factor is unproven. Nevertheless, it is now clear that from a therapeutic point of view, there is an imperative to reduce both the magnitude and the variability of CV risk factors.

This review focuses on the forms of BPV, evidence of BPV-associated long-term CV risks and evidence for therapeutic strategies to address BPV beyond levels of mean BP.

## ASSESSING BLOOD PRESSURE VARIABILITY

BP measurements are inexpensive, easily obtainable and can be collected repeatedly. Information based on more invasively obtained hemodynamic measurements is often considered more pertinent than information based on simple BP recordings. Historically beat-to-beat variability was measured invasively (intra-arterially) and was later succeeded by photoplethysmographic techniques. With the advent of ambulatory BP monitoring (ABPM) in the 1990s, noninvasive BP measured over a period of 24–48 h became feasible providing a robust tool to measure short-term BPV and gradually was widely yet unevenly adopted.

Short-term BPV reflects normal circadian rhythm-associated variability and within visit variability [9]. The nighttime dipping pattern of BP (normally ∼10–20%) is a physiological phenomenon but may be either absent ‘nondippers’ (<10% reduction), or reversed, and its classification is usually based on ABPM. A sharp morning rise in BP, referred to as ‘morning surge’, is another well studied physiological occurrence [10]. Medium-term BPV refers to variations in BP assessed between visits by office, home, or ABPM measurements over days, whilst long-term BPV is assessed using office BP (OBP) measurements and usually refers to visit-to-visit BPV in the context of multiple consecutive office visits [11,12] over months, seasons, years, or a lifetime. Effects of seasons on BP (higher during winter and lower during summer) have been systematically studied [13–15]. Long-term BPV is the type of variability with the strongest prognostic evidence and clinical value [11,12].

For long-term BPV assessments, office, home, and ABPM can all potentially be used for quantification. Long-term BPV from OBP can be obtained from medical records especially in healthcare settings that include electronic health records (EHR). However, these measurements are hugely variable, as they are affected by a multitude of factors including inconsistency of measurement techniques and the white coat effect. The intra-individual standard deviation (SD) of SBP (about 10–20 mmHg) between visits has been noted to be about 1.5–2 times greater than short-term variability assessed by measurements at a single visit. This then leads to the question of what is the best method for quantification of BPV?

## INDICES OF BLOOD PRESSURE VARIABILITY

The most commonly employed indices to quantify BPV are the SD and the coefficient of variation (CoV) of the BP measurements, especially for long term BPV quantification. The latter provides more reliable information by partially correcting for direct proportionality between the average BP and its variation. CoV provides an evaluation of the SD adjusted for average OBP, which is important as SD is closely correlated and dependent on average BP values [12]. However, SD and CoV are both influenced by acute stressors as mentioned above and day-night differences. Hence, other indices especially when evaluating short-term variability, have been proposed such as average real variability (ARV) which is the average of absolute differences between measurements and residual variability which is obtained by eliminating the slowest components of ABPM. Weighted SD of 24-h BP obtained from ABPM, allows quantification of short term 24 h BPV by excluding the contribution of nocturnal BP fall [11]. Variation independent of mean (VIM), obtained by transformation of SD, is derived from nonlinear regression analysis with the added advantage of not being correlated with mean BP itself. BPV quantified by VIM was shown to be associated with CV risk using large real-world data on visit-to-visit BPV derived from EHRs in a recent study [16] demonstrating the prognostic ability of BPV markers even when derived from nonstandardized BP reading.

## OFFICE BP VERSUS HOME BP VERSUS AMBULATORY BLOOD PRESSURE MONITORING

OBP measurement has been widely used in major hypertension outcome trials and the post hoc analyses of these trials have provided useful data on the importance of long-term BPV. The visit-to-visit BPV has been a well publicized long-term risk index and usually refers to OBP measured over a period of months-to-years.

The appropriate methodology for long-term BPV quantification lies in the optimal methodology for OBP measurement, which should be conducted according to current guidelines [17]. Nevertheless, the optimum and minimum requirements for a reliable assessment of long-term BPV have not been clearly defined. Clinical studies have used a variety of different and heterogeneous methods to measure and analyse office BPV data [12]. For example, typically three measurements of BP 1–2 min apart are recommended for optimal BP measurements, but it is unclear whether all three OBP measurements should be considered for BPV assessment as that would incorporate short-term variation within a long-term measurement. To what extent unattended OBP may provide more reliable and unbiased assessment of long-term BPV is uncertain.

ABPM when compared with OBP, is better in terms of both reproducibility and prognostic values of raised mean BP levels. However, undertaking ABPM at regular intervals is impractical for assessing long-term BPV. Do home BP (HBP) measurements undertaken over prolonged periods of time provide the most practical solution?

Procedures for HBP monitoring (HBPM) have been clearly defined by recent guidelines [18], and the average of HBP is well established as a risk factor for CV events. HBPM can be utilized to detect a wide range of BPV such as diurnal, day-by-day, seasonal, and long-term variability. Recent analysis of the Japan Morning Surge: Home blood pressure (J-HOP) study demonstrated that various home BPV measurements (calculated from 84 HBP readings measured 3 times in the morning and evening for 14 days) were not only feasible but were associated with CV event risk [19]. Morning-evening difference (ME-difference) using HBP was associated with CV events [20] and measures of day-by-day HBP variability (CoV; ARV; VIM) were independently associated with stroke events independent of the corresponding mean BP values [21]. Measures of home BPV (CoV, ARV, and VIM) were associated most strongly with CV risk in the winter [22]. However, in comparison with ABPM, HBP cannot provide robust measurements of night-time BPV. In a study of hypertensive adults on stable antihypertensive drug treatment, BPV was assessed with OBP, HBP, and ABPM in consecutive seasons without change in treatment [23]. BPV was quantified using the SD and CoV from OBP, HBP and daytime ABPM and was lower in summer than in winter (*P* < 0.01), whereas BPV derived from nighttime ABPM was unchanged. Further prospective data are needed to confirm seasonal variations of BP and BPV [23].

Thus, different methods for BP measurement (OBP, HBP, daytime ABPM, nighttime ABPM) provide different but complementary information on BPV [11,24–25]. Tables 1 and 2 summarize the BPV indices used in the literature and the method of obtaining BPV measurements respectively. The choice of BPV very likely will depend on the healthcare setting and is influenced by patient-physician preference.

**TABLE 1:** BPV indices (adapted from [12,24,25])

BPV index	Explanation	Formula/calculation	Significance
Standard deviation (SD) Weighted 24-h SD	Dispersion of BP readings from mean Weighted average of daytime and nighttime BP SD for separate durations of the day and nighttime periods	$SD=\sqrt{\sum {\left(xi-\mu \right)}^{2}}/N$, xi= each BP reading, μ is mean BP, N is number of readings Calculated by averaging the SD of these 2 subperiods	A high SD indicates both poor BP control and greater variability Adjustment for the two different time periods that have distinctly different physiological trends
Coefficient of variation (CoV)	Expression of SD as a percentage of the mean, providing a normalised measure of variability	$CoV=\left(SD/\mu \right)*100\%$, μ is mean BP	Comparison across different populations or time periods possible. A high CoV is an indicator of greater relative variability
Average real variability (ARV)	Quantification of the absolute average differences between consecutive BP readings	$ARV=\left(\sum \left(\left(xi-xi-1\right)/\left(N-1\right)\right)\right)$ where xi and xi-1 are consecutive BP readings.	Provides information on the short-term Fluctuations of blood pressure
Variation independent of mean (VIM)	Quantification of variation whilst completely removing the influence of the mean BP	$VIM=k\times SD/{\left(mean\right)}^{x}$, $k=mean{\left(mean\left(BP\right)\right)}^{x}$ x is obtained from fitting a nonlinear regression model (curve fitting of SD against mean) among the entire sample where SD=a times mean^x^	Provides variability independent of mean, however, depends on the distribution of BP within each cohort and value cannot be compared across populations
Residual SD	Quantification of calculating root mean-square of the residuals (i.e., difference between observed and predicted BP from regression.	First blood pressure changes over time (slope) defined as the beta coefficient for the linear regression of the blood pressure values is calculated. Residual mean square error denoted by the SD of the residuals from linear regression of the BP readings denotes the residual SD.	Though BPV derived is independent of mean, this is usually affected by the assumption that BP changes linearly over time

ARV, average real variability; BP, blood pressure; BPV, BP variability; CoV, coefficient of variation; SD, standard deviation; VIM, variation independent of mean.

**TABLE 2:** Methods of blood pressure variability measurement (adapted from [11,12,24])

Options for BPV measurement	BPV form	Indices	Advantages	Disadvantages
Office BP measurement	Long term	SD, CoV, ARV, VIM	Validated devices present, inexpensive, No extra resources needed	Masked hypertension and white coat hypertension and poor BP measurement technique possible
Home BP measurement	Long term, medium term, short term	SD, CoV, ARV, VIM	Validated devices present, inexpensive, Patient education needed	Calibration of devices is needed
Ambulatory BP measurement	Long term, medium term, short term	For long term and medium term BPV: SD, CoV, ARV, VIM For short term BPV: SD, CoV, VIM, ARV, Spectral analysis, 24-h weighted SD, 24-h weighted VIM	Validated devices available, patient education needed, good short-term BPV measure	Patient tolerance and adherence may impact utility. Repeated ABPM measurements for long term BPV resource intensive, not feasible in all healthcare service set ups
Continuous BP measurement	Very short term (beat to beat)	SD, CoV, ARV, spectral analysis	No discomfort to patients. Other health parameters can be measured simultaneously	Not validated, clinical application unclear

ARV, average real variability; BP, blood pressure; BPV, BP variability; CoV, coefficient of variation; SD, standard deviation; VIM, variation independent of mean.

## PATHOPHYSIOLOGY OF BLOOD PRESSURE VARIABILITY

BPV is expected to be higher in patients with hypertension versus normotension, in older versus younger patients and in patients with established EOD versus those without EOD. In common with other CV risk factors, measures of BPV were most predictive of EOD in younger and middle-aged rather than older subjects, and at lower values of mean SBP [26–29].

With age, the repeated cyclical stresses from ventricular ejection led to mechanical degeneration of the elastic lamellae and an increase in aortic stiffness i.e. a reduction in elasticity [30–32]. As might be expected, this process of ‘*normal vascular ageing*’ is accelerated in hypertensive individuals and those with elevated heart rates [32,34] and is affected by comorbidities such as T2DM and renal disease [33–37]. ‘Inflammaging’ – the occurrence of chronic low-grade inflammation occurring during aging is one of the key features of vascular aging associated with endothelial dysfunction. Bencivenga *et al.* comprehensively review the pathophysiological hallmarks of ageing including the molecular impact of senescence, whilst drawing parallels with the impact of BPV and propose BPV as a potential new marker of ageing [38].

The aetiology, pathophysiology, and prognosis associated with the different timescales of BPV are likely distinct from each other. Very short term and short-term BPV are influenced by the interaction of baroreceptor reflexes, sympathetic nervous system (SNS), renin–angiotensin–aldosterone system (RAAS), and nitric oxide (NO) release from the vascular endothelium. These physiological responses are strongly influenced by changes in the environment and behavioural disposition [11]. In animal studies excessive BPV leads to reduction in NO production from endothelial cells and remodelling of arteries promoting arterial stiffening [39]. Rizzoni *et al.* showed that increased BPV on ABPM was also associated with structural vascular changes assessed by minimal vascular resistance. They further demonstrated that high average ABPM values were associated with higher left ventricular (LV) mass [40]. Similarly, Schillaci *et al.* showed that arterial stiffness was strongly correlated with short-term BPV and also showed that the strength of the relationship varied depending on the statistical definition of BPV [41]. Increased arterial stiffness can damage small resistance vessels and lead to capillary rarefaction increasing men arterial pressure (MAP). Reduced vessel compliance is associated with compromised arterial baroreceptor sensitivity which in turn leads to abnormal BP variation, higher central BP and deranged circadian pattern of BP. Conversely, greater BPV could lead to greater mechanical stress in the vessels and arterial remodelling as shown in the Maastricht study [42]. Stiffer large arteries will magnify the BP changes due to variation in stroke volume, leading to greater absolute differences in SBP. Short-term BPV correlates with aortic pulse wave velocity (PWV) [40,41,42]. Longer-term BPV has also been associated with *progression* of carotid stiffening [43]. However, it is equally plausible that arterial stiffening leads to increased BPV [44]. BPV, hence can be considered both as a consequence and a cause of arterial stiffness and vascular dysfunction.

Acute rises in BP may be triggered by lifestyle and environmental factors including high salt diet, alcohol intake, temperature fluctuations, poor sleep quality and exercise, in susceptible individuals. The repeated insults of acute BP surges resulting from short term BPV, over time possibly lead to damage accrual leading to long-term BPV. Kario *et al.* hypothesize that the repeated synchronization of acute environmental or lifestyle triggers and physiological fluctuations in BP (independently associated with CV disease) might lead to resonance and amplification of short term variations in BP, in turn leading to higher long term BPV. According to this hypothesis, higher BPV would lead to acceleration of CV disease [45], resulting in greater incidence of CV events, which in turn predispose to further increase in BPV forming a vicious cycle.

A considerable proportion of what is perceived as long-term BPV may be the result of incorrect BP recording, either by patient or physician and this needs to be identified and rectified. Adherence to pharmacotherapy, intolerance to therapy, frequent dose escalation and/or de-escalation, prescription changes, pharmacokinetic factors such as drug half-life, and the time of the day of OBP measurement, may all have an impact on the short-term (within visit) and long-term BPV derived from OBP readings.

**FIGURE 1:**
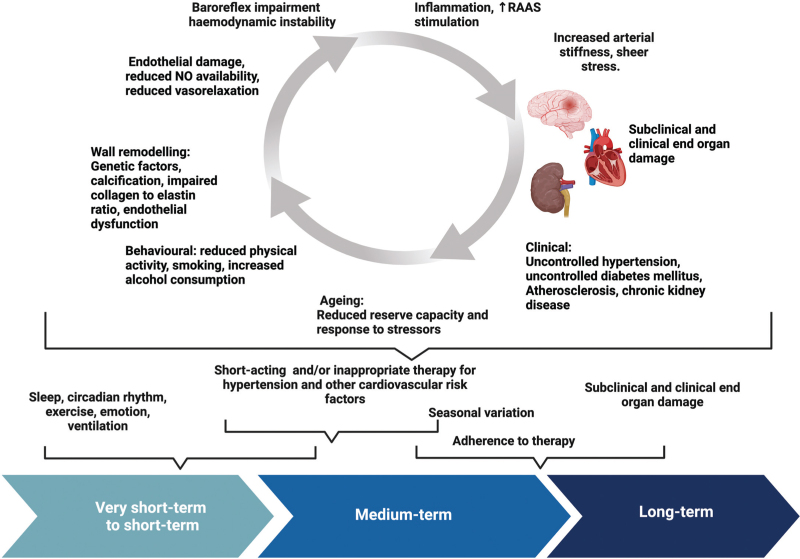
Pathophysiology of blood pressure variability (adapted from [12,38,45]) (created using Biorender.com). NO, nitric oxide; RAAS, renin–angiotensin–aldosterone system.

Figure 1 depicts these concepts visually.

## BLOOD PRESSURE VARIABILITY ASSOCIATION WITH CARDIOVASCULAR OUTCOMES

### Short-term blood pressure variability, cardiovascular outcomes and surrogates of cardiovascular outcomes

Several studies since 1990s have demonstrated the prognostic value of short-term BPV on CV events, including an early study using invasive arterial monitoring [2]. A systematic review investigating the role of 24-h BPV as a prognostic index confirmed that night dipping was associated with a lower risk of CV disease. The study also highlighted the divergent methodologies used to assess BPV in individual studies that affect pooling of results [46]. BPV in the face of an acute event such as stroke or myocardial function is known to be associated with a higher risk of future subsequent recurrence of CV events and mortality [47–49]. Mancia and Parati *et al.* reported that the variability of BP from ABPM predicted EOD over a follow-up period of 7.5 years [50]. Shortly thereafter, Otsuka and colleagues, reported that exaggerated circadian BP amplitude was an important predictor of ischaemic stroke and nephropathy over a follow-up period of 6 years even in normotensive subjects [51]. Table 1a, Supplemental Digital Content summarizes findings from key studies in the literature reporting the prognostic association of short-term BPV with CV outcomes.

Multiple small studies highlight the association of higher BPV (independent of mean) and worsening surrogates of end organ function including urine protein excretion [52], indices of myocardial structure (impacting LV function) [53,54], carotid intima thickness [55] and brain white matter hyperintensities [56]. However, a causative role of BPV cannot be assumed from any of these studies.

Other studies from Japan reported the prognostic values of home measured BPV [54,57]. BPV ascertained from HBPM may be largely referred to as ‘mid-term BPV’ as they generally do not indicate BPV over 24 h or within (visit) measurement BPV.

### Long term blood pressure variability and cardiovascular outcomes

The strength of evidence on the adverse impact of high BPV is greatest between higher long-term VVV in SBP and higher risks of all-cause mortality, CV mortality, and CV events [58–61]. A summary of the key studies is highlighted in Table 1b, Supplemental Digital Cntent.

ASCOT-BPLA [The Anglo-Scandinavian Cardiac Outcomes Trial-Blood Pressure Lowering Arm] was a RCT in hypertensive subjects, comparing two different treatment strategies, beta-blocker (BB); atenolol ± diuretic; bendroflumethiazide and calcium channel blocker (CCB); amlodipine ± angiotensin converting enzyme inhibitor (ACEi); perindopril (58). Mean BP readings were well controlled during the trial following randomisation, but there were small differences in achieved BP in favour of the amlodipine-based treatment by, on average, 2.7/1.4 mmHg. Preliminary analyses suggested that these small differences in BP did not account for differences in CV outcomes, which were in favour of the amlodipine-based treatment regimen for most CV outcomes, including mortality [59]. Subsequent re-analysis which included almost one million measurements of BP throughout the 5.5-year follow-up period showed that in-trial mean BP was a poor determinant of CV outcomes. On the other hand, visit-to-visit systolic BPV throughout the trial was strongly associated with stroke and coronary outcomes [25] Moreover, it was clear that there were substantial differences in the treatment effects on long-term BPV, with a gradual reduction throughout the trial with the amlodipine-based treatment regimen, contrasting with an initial rise in variability as participants were randomized to atenolol and, thereafter, as the trial progressed, a gradual fall presumably due to the introduction of second and third line drugs [62]. A number of other trials including the Antihypertensive and Lipid-Lowering Treatment to Prevent Heart Attack Trial (ALLHAT) [60], the ADVANCE trial [61] and systematic reviews and meta-analyses [63] reported similar observations on the importance of long-term VVV and associations with CV mortality outcomes beyond the effect of mean BP. The magnitude of these associations was similar to that between hypercholesterolaemia and CV disease. An approximately 5 mmHg increase in the SD of SBP was associated with a 10% increase in the risk of CV death [28].

In the legacy arm of ASCOT-BPLA data on hospitalizations and mortality were recorded over approximately 20 years of observation. Almost 5000 cardiovascular events, including approximately 3000 coronary events and 1000 strokes were recorded. In this long-term CV outcome study, there was a 20–25% increase in CV risk for each SD in SBP and among patients with well controlled BP (>5 years), a SD of 13 mmHg was associated with increased CV mortality [64]. The data, importantly, showed in those who achieved BP well within the normal range, higher levels of systolic BPV conferred substantial additional cardiovascular risk. These subjects had mean BP levels below the targets for treatment according to the existing guidelines (2000–2005) but have substantial residual CV risk. This population represent a target population for the treatment of BPV.

## BLOOD PRESSURE VARIABILITY AND RISK OF DEMENTIA

People with higher long-term BPV have an increased risk of stroke, and dementia. Plausibly, putative effects on the vasculature, leading to cerebral infarction, small vessel disease, or damage to the drainage pathways for cerebral amyloid might be induced by both short-and long term-BPV. Numerous cohort studies indicate that increased BPV correlates with an increased risk of dementia. A systematic review found that a unit increase in BPV resulted in an odds ratio (OR) of 1.25 for dementia, suggesting a strong association [65].

Another review of 27 studies demonstrated that higher BPV was associated with the presence of cerebral small vessel disease (OR, 1.27; 95% CI, 1.14–1.42), although with a high between study heterogeneity perhaps due to differences in methods of measurement between studies [66]. In a study of 54 cognitively un- or mildly- impaired adults, higher visit-to-visit BPV was modestly associated with higher levels of cerebrospinal fluid phosphorylated tau and lower levels of β-amyloid. In addition phosphorylated tau levels increased over time in carriers of APOE ε4 with higher BPV [67]. Interestingly, in the Rotterdam study, the hazard ratio in the highest compared with the lowest fifth of BPV was greater the longer the period between measurement and dementia [68] pointing towards plausible reverse causality.

Variability in multiple physiological measures is also associated with later cognitive impairment implying that physiological frailty or failure of homeostasis may be linked to the later risk of dementia, but not necessarily causal.

## BLOOD PRESSURE VARIABILITY AND CHRONIC KIDNEY DISEASE

Long-term visit-to-visit BPV is considered an independent determinant of renal deterioration, linked to faster decline in estimated glomerular filtration rate (eGFR) [69] and increased risk of CKD [70]. CKD itself contributes to increased BPV via multiple mechanisms including autonomic dysfunction, elevation in SNS, altered arterial stiffness and endothelial dysfunction, as renal function declines.

Data from the Korean National Health Insurance System (real-world data) revealed that patients in the highest quartile (quartile 4) of BPV had a 3.3-fold higher risk of end-stage renal disease (ESRD) compared to those in the lower quartiles (quartile 1 through 3) of BPV [71]. Another study utilizing 24-h ABPM found that BPV was independently associated with the initiation of dialysis or kidney transplantation [72]. A 1 mmHg increment in BPV was associated with a 5% increase in the risk of CKD based on a meta-analysis [73]. The similar effects of BPV on the brain and kidney may be due to similarity in the vascular structures within the two organs. The perforators that branch off from the intracranial arteries and in the glomerulus extend in a perpendicular manner with a sudden reduction in diameter render them susceptible to the effects of high BP and BPV. The correlation between cerebral white matter changes and microalbuminuria aligns with this hypothesis [73]. Other studies demonstrate that worsening renal function is associated with small increases in BPV across the spectrum of renal function [74] particularly in advanced CKD [75]. Whether reducing BPV will definitively prevent deterioration of renal function, needs further prospective evidence.

## ANTIHYPERTENSIVE DRUGS AND BLOOD PRESSURE VARIABILITY

Whilst there are no prospective RCT outcome data linking BPV reduction to decreased CV events, several posthoc analyses of RCTs and prospective observational studies provide evidence that certain antihypertensive therapies such CCBs and thiazide-like diuretics reduce BPV [11,76].

ASCOT clearly demonstrated that long term BPV was reduced by a CCB (amlodipine) in striking contrast with the betablocker atenolol which caused an increase in BPV [62]. Similarly, in a posthoc analysis of ALLHAT, long-term BPV was significantly different between the three randomization groups, being lowest in the CCB (amlodipine) group and highest in the ACEi (lisinopril) group, with the diuretic chlorthalidone group having an intermediate effect [77]. In a meta-analysis that included the ALLHAT trial and four other trials, long-term BPV was significantly lower in patients assigned to amlodipine than those assigned to enalapril (two trials), all ACEi (three trials), all active antihypertensive treatments [ACEi, a β-blocker atenolol, chlorthalidone, and an angiotensin-receptor blocker (ARB) losartan in five trials], and all comparators (plus a placebo group in one of the five trials) by a mean SD of 1.18 –1.55 mm Hg and a mean CoV of 0.79–1.08% [76]. Similar advantages were also observed for amlodipine over placebo, an ARB candesartan, and a thiazide diuretic indapamide slow-release, when BPV was derived from ABPM [78]. In another meta-analysis of 398 antihypertensive drug treatment trials with BP as endpoint, the interindividual SBP variation was lowest with CCBs than with all the other classes of antihypertensive drugs and placebo, regardless whether it was calculated as the pooled variation ratio of the comparison group SDs or as the pooled percentage increase in CoV [79]. A meta-analysis by Kollias *et al.*, considering the ASCOT, CAMELOT and ALLHAT trials, compared the change in visit-to-visit BPV and the accompanying change in outcome induced by treatment based on amlodipine versus treatment based on comparator drugs such atenolol, enalapril, chlorthalidone and lisinopril. This analysis showed that treatment with amlodipine was associated with a greater reduction in BPV than treatment with other drugs, and this reduction in BPV was associated with a reduction in adverse outcome [80].

The between-drug effect in lowering BPV was also observed in the recent Systolic Blood Pressure Intervention Trial (SPRINT) that demonstrated clinical outcome benefit in those patients assigned to intensive SBP control (120 mmHg) compared with those assigned to standard SBP control (140 mmHg) [81]. In a posthoc analysis of the SPRINT study, visit-to-visit systolic BPV, as assessed by residual SD and ARV using the data from 6 months to the end of follow-up, patients treated with CCBs had a lower variability than those treated with other classes of antihypertensive drugs by an average difference of −2 mmHg [82].

For short-term BPV, the most comprehensive evidence is from the Spanish ABPM registry. 13 765 patients treated with monotherapy, had significantly different BPV indices measured (*P* < 0.001) between patients on ARBs (*n* = 4722), ACEi (*n* = 4693), BB (*n* = 1825), CCBs (*n* = 1203) and diuretics (*n* = 1022). Ambulatory reading-to-reading BPV indices (24-h, daytime, and night-time SD, weighted SD and ARV), were lowest for CCBs followed by diuretics [83]. Other observational studies show both CCBs and diuretics significantly reduce home BPV resulting in secondary prevention of cardiovascular events, compared with RAAS inhibitors (RAASi), largely by impacting on the BP ‘morning surge’ [84].

Nonpharmacological therapy including dietary and physical training in patients with metabolic syndrome also has a beneficial effect on BPV associated with corresponding reduction in weight and PWV [85], despite a nonsubstantial impact on mean BP.

The Reducing blood pressure Variability in Essential hypertension with Ramipril vErsus Nifedipine GITS (REVERENT) trial was the first multicentre RCT designed to assess the impact of different drug classes on different types of BPV and EOD [86,87]. Individuals with high BPV (especially out-of-office) retained this classification even after BP lowering therapy which included long acting nifedipine and ramipril [84]. No difference was reported between the two therapeutic arms in short and mid-term BPV, while there was a greater reduction in visit-to-visit BPV with the CCB Nifedipine-GITS (gastrointestinal therapeutic system) than with Enalapril (in press). These partly negative results may be explained by the shorter half-life of GITS nifedipine in comparison with amlodipine, and similar to ramipril.

Overall, an ideal antihypertensive agent that lowers BP consistently throughout a 24-h period, maintains a normal circadian pattern of BP with easy to adhere to regimen, and the resulting decrease in BPV may be preferable for all patients with hypertension. Short-acting drugs are more likely to increase BPV assessed over 24-h due to fluctuation in plasma levels. Long-acting agents have more stable plasma concentrations and thus would be the natural choice for patients with high BPV. Antihypertensive treatment resulting in well controlled BP may lead to a reduction in BPV, independent of mean BP, initial office BP and 24-h SBP values, and thereby lead to CV risk reduction [88].

## SUMMARY

To conclude, BPV is complex in terms of mechanisms, measurement techniques, and indices. BPV, and in particular, long term BPV, is a confirmed determinant of CV risk mortality and morbidity based on available literature over and above the mean BP. However, knowledge gaps in the available prospective evidence, the heterogeneity of studies limiting the standardization of methods of BPV assessment and the absence of RCTs demonstrating that reduction of BPV improves outcomes independently of reduction of mean BP, are some of the reasons for the poor appreciation of the clinical importance of BPV. Most clinical guidelines discuss BPV but consider the evidence to be insufficient to recommend treatment for BPV alone.

The ESH working group on BP monitoring and cardiovascular variability published a position paper recently to guide and shape the future of the field of BPV with interesting proposals to address and build evidence [12]. A short review directed to physicians in clinical practice has also recently been published [89].

On such a background, the available studies showing that long-acting CCBs, such as amlodipine, are more efficacious in reducing short- and long-term BPV compared with other classes of antihypertensive drugs, suggest that, while waiting for further evidence, this class of antihypertensive drugs should be considered in patients with high short term or long-term visit-to-visit BPV.

Future goals will be to establish BPV as a new paradigm in the management of hypertensive patients. Efforts to design and conduct robust prospective evidence, with standardization of statistical tools for BPV will help utilize BPV as a prognostic marker for residual CV disease mortality and morbidity risk. Designing studies to explore the benefits of using pharmacological therapy targeted at patients with abnormal BPV and normal or near-normal BP will help clarify whether and how BPV could be considered as an additional therapeutic target for CV disease prevention.

## ACKNOWLEDGEMENTS

This review was based on a series of presentations given at a workshop on blood pressure variability sponsored by Viatris in February 2023. Viatris had no part in the writing of the review and its content is totally independent of the sponsor. PS wishes to acknowledge support from the Biomedical Research Centre Award to Imperial College NHS Healthcare Trust and is a former NIHR Senior Investigator.

### Conflicts of interest

S.K. received consultancy from Viatris & Mphar and funding from AstraZeneca; G.P. received honoraria from Viatris, Omron and Merck; S.B. received consultancy fees and honoraria from Abbott Vascular, Biotronik, Boston Scientific, Pfizer, Viatris, Shockwave, Inari, Imperative Care and Recor; G.B. received lecture fees from Viatris, Daiichi Sankyo, Neopharmed Gentil, Servier, Alfasigma, & Sanofi; K.K. received grants from Omron Healthcare Ltd, Fukuda Denshi Ltd & A&D Company Ltd; F.M. received consultany fees from Medtronic & Recor; GS received consultancy and lecture fees from AstraZeneca, Menarini, Sanofi-Aventis, Servier, and Viatris; J.-G.W. received honoraria from Novartis, Omron & Pfizer; W.W. received funding from Chief Scientists's Office, HDRUK & Alzheimer's Society and honoraria from Viatris; IW received grants from Viatris & Astra Zeneca, consultancy fees from Vaitris, Roche & Astra Zeneca and honoraria from Viatris; PS received funding, consultany fees, and honoraria from Viatris.

## Supplementary Material

Supplemental Digital Content

## Supplementary Material

Supplemental Digital Content
