# Supplementary material for: Blood pressure variability: a review
Source: J Hypertens. 2025 Mar 10;43(6):929–38. doi: 10.1097/HJH.0000000000003994 (PMC12052075; doi:10.1097/HJH.0000000000003994)
Supplement: Supplemental Digital Content [file jhype-43-0929-s001.docx]

**Supplementary Table 1a: Summary of evidence of key studies detailing cardiovascular associations of short-term blood pressure variability**

| **First author, year, reference** | **Study design (name), Population, (Number of participants)** | | **Aims/Objectives of the study** | **Blood pressure variability measures** | **Cardiovascular outcomes** | **Quantification of results and conclusions** | |
| --- | --- | --- | --- | --- | --- | --- | --- |
| Parati et al., 1987 ([1](#_ENREF_1)) | Cross-sectional study | Hospitalised patients with mild to severe essential hypertension assessed for end-organ damage (EOD)  (108) | Examine the relationship between end organ damage (EOD), mean blood pressure (BP) and BP variability (BPV) in ambulant subjects (24-hour intraarterial BP) | Standard deviation (SD) | EOD | For nearly any level of 24-h mean BP, subjects in whom the 24-h BP variability was low had a lower prevalence and severity of EOD than those in whom the 24-h BP variability was high (P<0.05) | Hypertension-dependent EOD relates to the extent of BPV.  Difficult to ascertain the acute effect of EOD itself on BPV in this study. |
| Frattola et al.,1993 ([2](#_ENREF_2)) | Prospective longitudinal observational study | Essential hypertension with variable severity (73) | Evaluation of prognostic value of 24-h BP average and BPV measured by Oxford technique by measuring EOD score at mean follow up 7.4 years | SD | EOD score | Variables determining EOD included BP level at follow up visit, initial level of EOD and BPV(SD of half hourly BP readings) | Provided the first longitudinal evidence of probable impact of BPV on EOD |
| Kikuya et al., 2000 ([3](#_ENREF_3)) | Prospective longitudinal observational study (Ohasama study) | General population, >40 years  (1542) | The association between the baseline BPV and heart rate variability measured using ambulatory blood pressure monitoring (ABPM) and subsequent cardiovascular (CV) mortality (mean period of follow up: 8.5 years) | SD | CV disease (CVD) mortality | Estimated Relative hazard (cox proportional model):  SD (day): Hazard ratio (HR) 2.51 (1.10 - 5.70); P<0.05  SD (night): 2.21 (1.11 - 4.43); P<0.05 | BPV and HR variability obtained every 30 minutes by ABPM were independent predictors for CV mortality |
| Sega et al., 2002 ([4](#_ENREF_4)) | Cross-sectional study (PAMELA study) | General population not receiving anti-hypertensive therapy  (3200) | Investigate the association between BPV and LVMI | SD | Left ventricular mass index (LVMI) | SBP variability: β coefficient =0.38; P<0.05  DBP variability: β=0.88; P<0.01 | Positive independent association between LVMI and BPV. |
| Björklund et al., 2004 ([5](#_ENREF_5)) | Prospective longitudinal observational study (ULSAM) | Elderly men enrolled in Uppsala  872 | To investigate the prognostic significance of 24-h ambulatory systolic (SBP), diastolic (DBP) and pulse pressure (PP), and BPV variability for cardiovascular morbidity in elderly men | SD | CV morbidity | Cox proportional hazard model  SD of SBP: 1.24 (1.07 - 1.42) | Variability of daytime SBP added important prognostic information, suggesting that 24-h ABPM may contribute to an improved risk assessment in elderly subjects. |
| Mancia et al., 2007 ([6](#_ENREF_6)) | Longitudinal analysis in PAMELA study | General population not receiving anti-hypertensive therapy, followed up 148 months  (2012) | Association between the baseline BPV and subsequent cardiovascular (CV) mortality | SD | CVD mortality | Cox proportional hazard model:  DBP variability: β coefficient = 0.175; P=0.002 | Short-term components of BPV accompanied by an increased CV risk and play a prognostic role |
| Verdecchia et al., 2007 ([7](#_ENREF_7)) | Prospective longitudinal cohort study  (PIUMA study) | Essential HTN, initially untreated  (2649) | To elucidate the prognostic value of BP variability in a large hypertensive population on CVD | SD | CVD | Cardiac events in high vs low BPV groups: SD of SBP (day): 1.45 v 0.72  SD of DBP (day): 1.29 v 0.91 SD of SBP (night): 1.58 v 0.62  SD of DBP (night): 1.32 v 0.85  Night-time SBP associated with 51% excess risk (P=0.024) | An enhanced variability in SBP during the night-time is an independent predictor of cardiac events in initially untreated hypertensive subjects. However, this was not independent of the mean BP. |
| Ozawa et al., 2009 ([8](#_ENREF_8)) | Cross-sectional study | Hypertensive participants  (111) | Examine a possible relationship between the BPV (on ABPM) and LVMI and between BPV and brachial-ankle pulse wave velocity (baPWV) | SD, CoV | LVMI, baPWV | Multivariable analysis  LVMI: β coefficient = 0.20; P=0.04, R^2^= 0.29 for SD of DBP (night-time); P=0.048 baPWV: β coefficient = 0.23; P=0.03, R^2^= 0.412 for SD of SBP (night-time) | Nighttime DBP variability in addition to absolute BP levels is significantly associated with LVMI and that nighttime SBP variability is significantly correlated with arterial wall stiffness as assessed by baPWV |
| Stolarz-Skrzypek et al., 2010 ([9](#_ENREF_9)) | Prospective longitudinal observational study (IDACO study) | Multiple populations included in ABPM dataset.  (11785) | Report risk estimates that were independently associated with the daytime and nighttime BP level. | SD, ARV | CVD mortality, CVD events | SBP: CVD mortality: HR: 1.17 (1.07 – 1.28); P<0.05 CVD events: HR: 1.07 (1.00–1.14); P<0.05 Cardiac events: 1.03 (0.94–1.13) NS Stroke events: 1.10 (1.00 - 1.21); P<0.05.  DBP: CVD mortality: HR: 1.21 (1.12 – 1.31); P<0.001 CVD events: HR: 1.07 (1.00–1.13); P<0.05 Cardiac events: 1.02 (0.94–1.11) NS Stroke events: 1.14 (1.05 - 1.23); P<0.05.  In fully adjusted model, that is when model included 24-h BP, ARV only explained 0.1% to the explained risk of a composite CV event | Short-term reading-to-reading BPV adds to the risk stratification for CV outcomes, however 24-h BP level was the main predictor for CVD. |
| Rothwell et al., 2010 ([10](#_ENREF_10)) | Post analysis of RCT (UK-TIA trial, ASCOT-BPLA) | Patients with previous transient ischaemic attack (UK-TIA); HTN (treated) in ASCOT-BPLA  (1905) | To investigate whether the effects of these drugs on variability in blood pressure might explain these disparities in effect on stroke risk. For short-term variability assessment, drug effects were not studied | SD, CoV, VIM | Stroke events | SD daytime SBP on ABPM: HR per SD increase=1·29, 1·08–1·55, (p=0·007). Daytime coefficient of variation of SBP on ABPM was most predictive in patients with lower (<median) mean daytime SBP (HR per SD increase: 1·42, 1·18–1·71). | BPV from ABPM was weaker predictor of vascular events in comparison to visit to visit variability |
| Johansson et al., 2012 ([11](#_ENREF_11)) | Prospective Longitudinal observational study (The Finn-Home study) | Adults aged 45-74 years followed up over 7.8 years  (1866) | Assess the prognostic value of variability in home BP (HBP) and heart rate in a general population | SD | CVD events | Morning-evening: SBP variability 1.04 (1.01 - 1.07)  DBP variability 1.10 (1.05 - 1.15) Morning Day-day: SBP variability: 1.04 (1.00 - 1.07)  DBP variability: 1.10 (1.04 - 1.16) | Greater BPV HBP and heart rate are independent predictors of CV events. |
| Eguchi et al., 2012 ([12](#_ENREF_12)) | Prospective longitudinal Observational study | Asymptomatic patients being assessed for hypertension (457) | To clarify the impacts of clinic and ambulatory BPV in predicting CVD | SD | CVD events | SD of nighttime SBP: Stroke, myocardial dysfunction and sudden cardiac deaths: 2.21 (1.08- 4.53); P<0.03 | Visit-to-visit BPV and ambulatory BPV are separately useful in predicting cardiovascular outcomes. |
| Palatini et al., 2014([13](#_ENREF_13)) | International registry study: ABPM-based observational study | Ambulatory BP International Study: 8 prospective studies, with mean follow up for 5.5 years  (7112) | Investigate whether short term BPV, predicts CV events and mortality in hypertension | SD | CVD mortality and event | CVD mortality: 1.55 (1.05 - 2.28) for rise in SD of SBP (night); (P=0.028) CVD events: 1.41 (1.18 - 1.68) for SD of SBP (night); (P=0.0001) CVD mortality 2.32 (1.48 - 3.65) for SD of DBP (night); P=0.0003 CVD events 1.48 (1.22 - 1.78) for SD of DBP (night); P<0.0001. night-time SBP SD of >12.2 mmHg was associated with 41% greater risk of CV events, 55% greater risk for CV death. For DBP SD of >7.9 mmHg was associated with 48% greater risk of CV events, 132% greater risk for CV death | Addition of BPV measurements to models of long-term outcomes improved the ability to stratify appropriately patients with hypertension. |
| Mena et al., 2014 ([14](#_ENREF_14)) | Prospective longitudinal observational study (IDACO study) | Copenhagen cohort (subset of IDACO) (Discovery analysis: 2311)  (Test analysis: 5353) | To determine the minimum number of BP readings required to compute ARV without loss of prognostic information | ARV | CVD mortality  Cardiac mortality Cerebrovascular events (fatal and non-fatal) | Diastolic ARV: CV mortality: HR = 1.19 Cardiac mortality: HR = 1.19 Cerebrovascular events (fatal and non-fatal): HR = 1.16 (P<0.01) Systolic ARV: CVD mortality HR =1.17 Cardiac mortality HR = 1.24 (P<0.01) | 48 BP readings over 24 hours were needed to adequately compute ARV without meaningful loss of prognostic information. |

ABPM: Ambulatory Blood Pressure Monitoring, ARV: average real variability, ASCOT-BPLA: Anglo-Scandinavian Cardiac Outcomes Trial Blood Pressure Lowering Arm, baPWV: brachial-ankle pulse wave velocity BP: blood pressure, BPV: blood pressure variability, CV: cardiovascular, CVD: Cardiovascular disease, CoV: coefficient of variation, DBP: diastolic blood pressure, EOD: end-organ damage, HR: hazard ratio, IDACO: International Database on Ambulatory Blood Pressure in Relation to Cardiovascular Outcomes, Left ventricular mass index (LVMI), PAMELA: Pressioni Arteriose Monitorate E Loro Associazioni, PIUMA: Progetto Ipertensione Umbria Monitoraggio Ambulatoriale*,* SBP: systolic blood pressure, SD: standard deviation, UK-TIA: United Kingdom-transient ischaemic attack, ULSAM: Uppsala Longitudinal Study of Adult Men, VIM: Variation independent of mean

Please note that this is not an exhaustive list of studies that demonstrate association between short-term BPV and CV mortality and morbidity.

1. Parati G, Pomidossi G, Albini F, Malaspina D, Mancia G. Relationship of 24-hour blood pressure mean and variability to severity of target-organ damage in hypertension. Journal of hypertension. 1987;5(1):93-8.

2. Frattola A, Parati G, Cuspidi C, Albini F, Mancia G. Prognostic value of 24-hour blood pressure variability. Journal of hypertension. 1993;11(10):1133-7.

3. Kikuya M, Hozawa A, Ohokubo T, Tsuji I, Michimata M, Matsubara M, et al. Prognostic significance of blood pressure and heart rate variabilities: the Ohasama study. Hypertension (Dallas, Tex : 1979). 2000;36(5):901-6.

4. Sega R, Corrao G, Bombelli M, Beltrame L, Facchetti R, Grassi G, et al. Blood pressure variability and organ damage in a general population: results from the PAMELA study (Pressioni Arteriose Monitorate E Loro Associazioni). Hypertension (Dallas, Tex : 1979). 2002;39(2 Pt 2):710-4.

5. Bjorklund K, Lind L, Zethelius B, Berglund L, Lithell H. Prognostic significance of 24-h ambulatory blood pressure characteristics for cardiovascular morbidity in a population of elderly men. Journal of hypertension. 2004;22(9):1691-7.

6. Mancia G, Bombelli M, Facchetti R, Madotto F, Corrao G, Trevano FQ, et al. Long-Term Prognostic Value of Blood Pressure Variability in the General Population. Hypertension (Dallas, Tex : 1979). 2007;49(6):1265-70.

7. Verdecchia P, Angeli F, Gattobigio R, Rapicetta C, Reboldi G. Impact of blood pressure variability on cardiac and cerebrovascular complications in hypertension. American journal of hypertension. 2007;20(2):154-61.

8. Ozawa M, Tamura K, Okano Y, Matsushita K, Ikeya Y, Masuda S, et al. Blood pressure variability as well as blood pressure level is important for left ventricular hypertrophy and brachial-ankle pulse wave velocity in hypertensives. Clinical and experimental hypertension (New York, NY : 1993). 2009;31(8):669-79.

9. Stolarz-Skrzypek K, Thijs L, Richart T, Li Y, Hansen TW, Boggia J, et al. Blood pressure variability in relation to outcome in the International Database of Ambulatory blood pressure in relation to Cardiovascular Outcome. Hypertension research : official journal of the Japanese Society of Hypertension. 2010;33(8):757-66.

10. Rothwell PM, Howard SC, Dolan E, O'Brien E, Dobson JE, Dahlof B, et al. Prognostic significance of visit-to-visit variability, maximum systolic blood pressure, and episodic hypertension. Lancet (London, England). 2010;375(9718):895-905.

11. Johansson JK, Niiranen TJ, Puukka PJ, Jula AM. Prognostic Value of the Variability in Home-Measured Blood Pressure and Heart Rate. Hypertension (Dallas, Tex : 1979). 2012;59(2):212-8.

12. Eguchi K, Hoshide S, Schwartz JE, Shimada K, Kario K. Visit-to-visit and ambulatory blood pressure variability as predictors of incident cardiovascular events in patients with hypertension. American journal of hypertension. 2012;25(9):962-8.

13. Palatini P, Reboldi G, Beilin LJ, Casiglia E, Eguchi K, Imai Y, et al. Added predictive value of night-time blood pressure variability for cardiovascular events and mortality: the Ambulatory Blood Pressure-International Study. Hypertension (Dallas, Tex : 1979). 2014;64(3):487-93.

14. Mena LJ, Maestre GE, Hansen TW, Thijs L, Liu Y, Boggia J, et al. How many measurements are needed to estimate blood pressure variability without loss of prognostic information? American journal of hypertension. 2014;27(1):46-55.
