# Supplementary material for: Blood pressure variability: a review
Source: J Hypertens. 2025 Mar 10;43(6):929–38. doi: 10.1097/HJH.0000000000003994 (PMC12052075; doi:10.1097/HJH.0000000000003994)
Supplement: Supplemental Digital Content [file jhype-43-0929-s002.docx]

**Table 1b: Summary of evidence of studies detailing cardiovascular associations of long-term blood pressure variability**

| **First author, year, reference** | **Study Design (name)**  **Population, (number of participants)** | | **Aims and objectives of the study** | **Blood pressure variability measures** | **Cardiovascular outcomes** | **Quantification of results and conclusions** | | |
| --- | --- | --- | --- | --- | --- | --- | --- | --- |
| Kikuya et al., 2008 ([1](#_ENREF_1)) | Prospective longitudinal Observational study (Ohasama study) | General Population  (2455) | Investigate the prognostic value of day-by-day home blood pressure variability (BPV) in predicting cardiovascular (CV) mortality | Standard deviation (SD) Coefficient of variation (CoV) | CV disease (CVD) mortality, Stroke mortality, | Cox regression analysis: Systolic blood pressure (SBP) variability of 1 SD: CVD mortality: Hazard ratio (HR): 1.20 (1.02 - 1.40); P≤0.05 Stroke mortality: HR: 1.38 (1.12 - 1.72); P≤0.01 | Day-by-day BPV and heart rate variability by self-measurement at home provides useful clinical information for assessing CV risk. |  |
| Rothwell et al., 2010 ([2](#_ENREF_2)) | Post-hoc analysis from randomised control trial (RCT)  (UK-TIA trial, ASCOT-BPLA) | Patients with previous transient ischaemic attack (TIA; UK-TIA); hypertension (in ASCOT-BPLA  (2011) | To establish the prognostic significance of visit-to-visit variability (VVV) in blood pressure (BP), and maximum SBP in treated patients over seven visits | SD, CoV, Variability independent of mean (VIM) | CVD event | Top-decile of SD SBP:  Stroke events: TIA cohort: 6.22 (4.16 - 9.29); P<0.0001.  Hypertension: ASCOT-BPLA cohort: 3.25 (2.32 - 4.54); P<0.0001 | SBP VVV and maximum SBP are strong predictors of stroke, independent of mean SBP. |  |
| Poortvliet et al., 2012 ([3](#_ENREF_3)) | Post-hoc analysis from RCT (PROSPER trial) | Elderly participants with pre-existing vascular disease or at high risk of atherosclerosis, due to hypertension, smoking or diabetes.  (4819 (short-term follow-up)) (1808 (long-term follow-up)) | To establish whether VVV in BP in older patients is associated with increased risk of incident CVD. | SD, CoV | CVD mortality, Stroke events, CHD events, CVD events | HR: SBP variability: CVD mortality 1.2 (1.1 - 1.4) DBP variability: CVD mortality 1.4 (1.1 - 1.7) Coronary events 1.5 (1.2 - 1.8) Heart failure hospitalisation 1.4 (1.1 - 1.8) | VVV in diastolic blood pressure (DBP) is more strongly associated with vascular or total mortality than is systolic pressure variability in older high-risk subjects. |  |
| Carr et al., 2012 ([4](#_ENREF_4)) | Post-hoc analysis of RCT (MRC Elderly trial) | Hypertensive patients with a mean SBP of 160–209 mmHg (with DBP <115 mmHg) was required for entry.  (4396) | Assess the impact of BPV associated with drugs used for treatment in the study on CV risk | Maximum BP, trend adjusted SD of successive BP measurement referred to as root successive variance (RSV) | CVD events (stroke and coronary heart disease (CHD)) | An increase in 1 SD (Similar for RSV) of SBP: Stroke events: 1.15 (1.01-1.31). CHD events: 1.16 (1.00 - 1.36) | Individual risk characterization could be augmented with additional prognostic information, besides current SBP, including current diastolic pressure, temporal variability over and above general trends and historical measurements. |  |
| Hsieh et al., 2012 ([5](#_ENREF_5)) | Prospective longitudinal cohort study | Patients with type 2 diabetes mellitus (T2DM)  (2161) | The association between CV mortality and blood pressure parameters in patients with T2DM | SD, CoV | CVD mortality | VVV in pulse pressure (PP) predicted CV mortality: HR: 1.139 (1.030 - 1.258); P=0·01 | VVV in BP was significantly associated with all-cause mortality and CV mortality independent of mean BP in patients with T2DM. |  |
| McMullan et al., 2013([6](#_ENREF_6)) | Prospective cohort study (African American Study of Kidney disease (AASK) study) | African Americans, aged 18 to 70 years, with hypertensive CKD  (908) | Association of VVV in SBO with overall mortality, CV mortality, CV events, and renal events among individuals enrolled in the AASK trial | SD calculated SBP over 5 visits occurring 3-12 months after randomisation | CVD mortality | Higher VVV in SBP associated with CV mortality.  HR: 4.91 (1.12 - 21.50) | In African Americans with CKD, SBV is strongly and independently associated with overall and CV mortality. |  |
| Kawai et al., 2013 ([7](#_ENREF_7)) | Observational Cohort study (NOAH) | Patients with essential hypertension  (485) | Assess association of VVV of SBP correlation with CVD, CV mortality, statin use, pulse wave velocity (PWV), left ventricular mass index (LVMI), plaque score, estimated glomerular filtration rate (eGFR). Quantify the level of BPV that is associated with CVD and CV mortality | SD | CVD events | SD of SBP≥8.1predicted CV events and SD of SBP≥ 13.7 predicted mortality | VVV in SBP is significantly associated with systemic atherosclerotic change, incidence of CVD, and mortality rate. Altered arterial functions, such as macrovascular atherosclerosis and vascular resistance, are responsible for the correlations between SBP VVV and incidence of CVD. |  |
| Hata et al., 2013 ([8](#_ENREF_8)) | Post-hoc analysis of RCT (ADVANCE trial) | Patients with T2DM and history of major macro-/microvascular disease  (8811) | Assess the effects VVV in SBP and maximum SBP on the vascular outcomes | SD, CoV | Microvascular and macrovascular complications of T2DM | BPV in the highest tenth-decile associated with macrovascular events: 1.54 (0.99 – 2.39)  microvascular events: 1.84 (1.19 – 2.84) | VVV in SBP and maximum SBP were independent risk factors for macrovascular and microvascular complications in T2DM |  |
| Kostis et al., 2014 ([9](#_ENREF_9)) | Post-hoc analysis of RCT (SHEP trial) | Elderly patients with isolated systolic hypertension  (4320) | To assess if relationship of high VVV BP and long‐term CV mortality and if it is associated with inconsistent (visit to visit) adherence to medications | Sum of squared deviations between each SBP and trend-predicted SBP (rSSR), variance of the absolute values of the second differences between successive daily SBP values (VABS2), VIM | CVD mortality | rSSR 1.0086 (1.0026 – 1.015); P<0.01 VABS2 1.001 (1.0002 – 1.001); P<0.01 | SBP VVV predicted CV death at 17 years of follow‐up. Adherence was not a factor detected to impact this relationship in this study |  |
| Blacher et al., 2015 ([10](#_ENREF_10)) | Post-hoc analysis of RCT (SU.FOL.OM3) | Participants of with coronary or cerebral ischaemic acute event 1-12 months prior to inclusion  (2157) | To establish if BPV measurement is of additive value, in terms of CV risk assessment strategies | SD, CoV | CVD events | CoV of SBP: OR for major CV event 1.23 (1.04 - 1.46); P=0.016 | BPV has an independent prognostic value in the prediction of major CV events; but improvement in the prediction model was quite modest. In favour of BPV acting as an integrator of CV risk than acting as a robust independent CV risk factor in this high-risk population. |  |
| Rossignol et al., 2015([11](#_ENREF_11)) | Post-hoc analysis of RCT (HEAAL trial) | Participants with heart failure with reduced ejection fraction (HFrEF)  (3732) | Assess the association between VVV in BP and outcomes in chronic heart failure (CHF) patients, | SD, CoV, ARV | CVD event (hospitalisation for worsening heart failure) | Higher VVV in SBP was associated with poorer outcomes: HR: 1.023 (1.013 - 1.034); P <0.0001 | VVV in SBP was associated with poorer CV outcomes. |  |
| Arashi et al., 2015 ([12](#_ENREF_12)) | Post-hoc analysis of RCT (HIJ-CREATE) | Hypertension with coronary artery disease (CAD) at baseline (1734) | Clarify the prognostic significance of VVV in systolic BP on subsequent CVD/ major adverse cardiac event (MACE) in hypertensive patients with CAD | SD, CoV, VIM | CVD events | VVV in SBP was not associated with subsequent MACE (SD: p=0.263) | In hypertensive patients with CAD, inadequate BP control is a strong predictor of subsequent MACE, whereas VVV of SBP is not. |  |
| Rakugi et al., 2015 ([13](#_ENREF_13)) | Sub-analysis of RCT (COLM trial) | Participants >65 years of age with hypertension and/or risk factors for CVD in the study with office BP measured at least 3 times  (4876) | Compare VVV of BP between age groups and between two treatment combinations, that is, the angiotensin II receptor blocker, olmesartan combined with a calcium channel blocker (CCB), or a diuretic and to investigate the effect of VVV of BP on CV events in elderly hypertensive patients. | SD, VIM, ARV | CVD mortality and morbidity | SD of SBP was associated with primary composite end point in both elderly and very elderly categories of participants. Olmesartan and CCB had a lower SBP SD compared with olmesartan and diuretic | VVV of SBP may mediate the preferable effect of combination of angiotensin II receptor blocker along with CCB on CV events in the very elderly and isolated systolic hypertensive patients. |  |
| Muntner et al., 2015 ([14](#_ENREF_14)) | post-hoc analysis of RCT (ALLHAT trial) | Hypertension, participants without CVD events during the first 28 months of follow-up  25814 | Association of VVV long-term SBP and DBP with CVD and mortality across 7 visits between 6-28 months following enrolment. | SD, SDIM, ARV | CVD events (fatal CHD and non-fatal MI, stroke, heart failure) | HR comparing highest and lowest quintile of SD SBP:  Fatal CHD or non-fatal MI: 1.30 (1.06 - 1.59); P=0.006  Heart failure: 1.25 (0.97 - 1.61); P=0.084 Stroke :1.46 (1.06 - 2.01); P=0.0013 | Higher VVV of SBP is associated with an increased risk for CVD and mortality. |  |
| Gosmanova et al., 2016([15](#_ENREF_15)) | Retrospective cohort study | US veterans with and without hypertension, normal kidney function with ≥8 measurements  2865157 | Association of increased SBP VVV and all-cause mortality, CV events, and end-stage renal disease (ESRD) | SD | CV events, CV mortality | CHD: SD quartile 2: 2.11 (2.02 - 2.19)  SD quartile 3: 3.59 (3.45 - 3.72)  SD quartile 4: 5.92 (5.70 - 6.14)   Stroke: SD quartile 2: 2.05 (1.95 - 2.14)  SD quartile 3: 3.63 (3.47 - 3.79)  SD quartile 4: 6.60 (6.32 - 6.89)  ESRD:  SD quartile 2: 1.33 (1.10 - 1.14)  SD quartile 3: 2.56 (2.16 - 3.03)  SD quartile 4: 10.59 (9.02 – 12.43) | Higher BPV in individuals with and without hypertension was associated with increased risks of all-cause mortality, CHD, stroke, and ESRD. |  |
| Tedla et al., 2017)([16](#_ENREF_16)) | Longitudinal cohort study (MESA) | Participants free of clinical CVD at baseline Multi-ethnic Study of atherosclerosis  (1122) | Investigate the association between long-term SBP variability and ten-year percent change in arterial stiffness | SD, CoV, VIM | Carotid artery stiffness progression (Distensibility Coefficient, (DC); Young's Elastic Modulus, (YEM)) | At 10 years of follow up, individuals in the 5^th^ quantile of BPV had higher decline in DC: −9.84 (−16.96 to −2.72); P≤0.01 and a higher progression in YEM.  YEM: 27.53 (15.79 - 39.27); P≤0.001 | Higher long-term SBP variability may be a risk factor for arterial stiffness progression independent of mean BP |  |
| Mehlum et al., 2018 ([17](#_ENREF_17)) | Post-hoc analysis of RCT (VALUE) | Participants with hypertension at high risk of cardiac disease with BP from ≥3 visits after 6 months and no events during the 6 months  (13803) | To assess if BPV was associated with increased risk of CV events and death in hypertensive patients at different risk levels | SD | CVD events, CV mortality | Highest quantile of SD SBP had increased risk of CV events: 2.1 (1.7 - 2.4); P<0.0001. a 5 mm increase in SD SBP is associated with a 10% increase in risk of death | Higher VVV SBP is associated with increased risk of CV events in patients with hypertension, irrespective of baseline risk of CV events. |  |
| Ebinger et al., 2022 ([18](#_ENREF_18)) | Retrospective cohort study | Patients followed in clinical practice over six years  (42482) | To determine if BPV is associated with adverse CV outcomes using clinically generated data from the electronic health record (EHR) | VIM (SBP, DBP) | CVD mortality, CVD events: MI, HF, Stroke; individual and composite outcomes | Composite outcome: VIM SBP: 1.22 (1.17 - 1.28)  VIM DBP: 1.24 (1.19 - 1.30) | VIM derived from clinically generated data remains associated with adverse CV outcomes and represents a risk marker beyond mean BP, including in important demographic and clinical subgroups. |  |
| Gupta et al., 2024 ([19](#_ENREF_19)) | Post-hoc analysis of RCT (ASCOT-Legacy arm) | Hypertension (treated) in ASCOT-Legacy cohort  (2156) | Determine the CV impact of mean long-term SBP control and long-term BPV during the trial, and amongst those allocated to amlodipine- and atenolol-based treatment | SD, VIM | CVD events CVD mortality | BPV is a predictor of CV events; HR: per 5 mmHg:1.22 (95% CI:1.18 - 1.26); P<0.001 and predicted events in patients with well-controlled BP. Amlodipine based treatment significantly reduced CV events. | Systolic BPV is a strong predictor of CV outcome, even in those with controlled SBP. Long term benefits of amlodipine compared to atenolol appear related to effect on systolic BPV |  |

AASK: African American Study of Kidney disease, ALL-HAT: Antihypertensive and Lipid-Lowering Treatment to Prevent Heart Attack Trial ASCOT-BPLA: Anglo-Scandinavian Cardiac Outcomes Trial-blood pressure lowering arm, BP: blood pressure, BPV: blood pressure variability, calcium channel blocker (CCB), CHD: coronary heart disease, COLM: The Combination of OLMesartan and a calcium channel blocker (CCB) or a diuretic, in Japanese elderly hypertensive patients, CV: cardiovascular, CVD: Cardiovascular disease, CoV: coefficient of variation, DBP: diastolic blood pressure, DC: Distensibility Coefficient, ELSA: European Lacidipine Study on Atherosclerosis, ESRD: end-stage renal disease, HEAAL: Heart failure Endpoint evaluation of Angiotensin II Antagonist Losartan, HFrEF: heart failure with reduced ejection fraction, HR: hazard ratio, MACE: major adverse cardiac event, MESA: Multi-ethnic Study of atherosclerosis, NOAH: Non-Invasive Atherosclerotic Evaluation in Hypertension*,* PWV: pulse wave velocity, PROSPER: Prospective Study of Pravastatin in the Elderly at Risk, RCT: Randomised control trial, rSSR: Sum of squared deviations between each BP and trend-predicted BP, RSV: root successive variance, SBP: systolic blood pressure, SD: standard deviation, SHEP: Systolic Hypertension in the Elderly Program, T2DM: type 2 diabetes mellitus, UK-TIA: United Kingdom-transient ischaemic attack, VABS2: variance of the absolute values of the second differences between successive daily SBP values VALUE: The Valsartan Antihypertensive Long-term Use Evaluation, VIM: Variation independent of mean, YEM: Young's Elastic Modulus

Please note that this is not an exhaustive list of studies that demonstrate association between long term BPV and CV mortality and morbidity.

References

1. Kikuya M, Ohkubo T, Metoki H, Asayama K, Hara A, Obara T, et al. Day-by-Day Variability of Blood Pressure and Heart Rate at Home as a Novel Predictor of Prognosis. Hypertension (Dallas, Tex : 1979). 2008;52(6):1045-50.

2. Rothwell PM, Howard SC, Dolan E, O'Brien E, Dobson JE, Dahlof B, et al. Prognostic significance of visit-to-visit variability, maximum systolic blood pressure, and episodic hypertension. Lancet (London, England). 2010;375(9718):895-905.

3. Poortvliet RK, Ford I, Lloyd SM, Sattar N, Mooijaart SP, de Craen AJ, et al. Blood pressure variability and cardiovascular risk in the PROspective Study of Pravastatin in the Elderly at Risk (PROSPER). PLoS One. 2012;7(12):e52438.

4. Carr MJ, Bao Y, Pan J, Cruickshank K, McNamee R. The predictive ability of blood pressure in elderly trial patients. Journal of hypertension. 2012;30(9):1725-33.

5. Hsieh YT, Tu ST, Cho TJ, Chang SJ, Chen JF, Hsieh MC. Visit-to-visit variability in blood pressure strongly predicts all-cause mortality in patients with type 2 diabetes: a 5.5-year prospective analysis. European journal of clinical investigation. 2012;42(3):245-53.

6. McMullan CJ, Bakris GL, Phillips RA, Forman JP. Association of BP Variability with Mortality among African Americans with CKD. Clinical Journal of the American Society of Nephrology. 2013;8(5).

7. Kawai T, Ohishi M, Ito N, Onishi M, Takeya Y, Yamamoto K, et al. Alteration of vascular function is an important factor in the correlation between visit-to-visit blood pressure variability and cardiovascular disease. J Hypertens. 2013;31(7):1387-95; discussion 95.

8. Hata J, Arima H, Rothwell PM, Woodward M, Zoungas S, Anderson C, et al. Effects of Visit-to-Visit Variability in Systolic Blood Pressure on Macrovascular and Microvascular Complications in Patients With Type 2 Diabetes Mellitus. Circulation. 2013;128(12):1325-34.

9. Kostis JB, Sedjro JE, Cabrera J, Cosgrove NM, Pantazopoulos JS, Kostis WJ, et al. Visit-to-visit blood pressure variability and cardiovascular death in the Systolic Hypertension in the Elderly Program. Journal of clinical hypertension (Greenwich, Conn). 2014;16(1):34-40.

10. Blacher J, Safar ME, Ly C, Szabo de Edelenyi F, Hercberg S, Galan P. Blood pressure variability: cardiovascular risk integrator or independent risk factor? Journal of human hypertension. 2015;29(2):122-6.

11. Rossignol P, Girerd N, Gregory D, Massaro J, Konstam MA, Zannad F. Increased visit-to-visit blood pressure variability is associated with worse cardiovascular outcomes in low ejection fraction heart failure patients: Insights from the HEAAL study. International journal of cardiology. 2015;187:183-9.

12. Arashi H, Ogawa H, Yamaguchi J, Kawada-Watanabe E, Hagiwara N. Impact of visit-to-visit variability and systolic blood pressure control on subsequent outcomes in hypertensive patients with coronary artery disease (from the HIJ-CREATE substudy). The American journal of cardiology. 2015;116(2):236-42.

13. Rakugi H, Ogihara T, Saruta T, Kawai T, Saito I, Teramukai S, et al. Preferable effects of olmesartan/calcium channel blocker to olmesartan/diuretic on blood pressure variability in very elderly hypertension: COLM study subanalysis. Journal of hypertension. 2015;33(10):2165-72.

14. Muntner P, Whittle J, Lynch AI, Colantonio LD, Simpson LM, Einhorn PT, et al. Visit-to-Visit Variability of Blood Pressure and Coronary Heart Disease, Stroke, Heart Failure, and Mortality. Ann Intern Med. 2015;163(5):329-38.

15. Gosmanova EO, Mikkelsen MK, Molnar MZ, Lu JL, Yessayan LT, Kalantar-Zadeh K, Kovesdy CP. Association of Systolic Blood Pressure Variability With Mortality, Coronary Heart Disease, Stroke, and Renal Disease. Journal of the American College of Cardiology. 2016;68(13):1375-86.

16. Tedla YG, Yano Y, Carnethon M, Greenland P. Association Between Long-Term Blood Pressure Variability and 10-Year Progression in Arterial Stiffness. Hypertension (Dallas, Tex : 1979). 2017;69(1):118-27.

17. Mehlum MH, Liestol K, Kjeldsen SE, Julius S, Hua TA, Rothwell PM, et al. Blood pressure variability and risk of cardiovascular events and death in patients with hypertension and different baseline risks. European heart journal. 2018;39(24):2243-51.

18. Ebinger JE, Driver M, Ouyang D, Botting P, Ji H, Rashid MA, et al. Variability independent of mean blood pressure as a real-world measure of cardiovascular risk. EClinicalMedicine. 2022;48:101442.

19. Gupta A, Whiteley WN, Godec T, Rostamian S, Ariti C, Mackay J, et al. Legacy benefits of blood pressure treatment on cardiovascular events are primarily mediated by improved blood pressure variability: the ASCOT trial. European heart journal. 2024;45(13):1159-69.
